# Bone Quality Indices Correlate with Growth Hormone Secretory Capacity in Women Affected by Weight Excess: A Cross-Sectional Study

**DOI:** 10.3390/jcm13175064

**Published:** 2024-08-27

**Authors:** Elena Gangitano, Maria Ignazia Curreli, Orietta Gandini, Davide Masi, Maria Elena Spoltore, Lucio Gnessi, Carla Lubrano

**Affiliations:** Department of Experimental Medicine, Sapienza University of Rome, 00161 Rome, Italy

**Keywords:** bone mineral density, dual-energy X-ray absorptiometry, DXA, growth hormone, obesity, overweight, gender medicine, women, female

## Abstract

**Background/Objectives:** Obesity can be associated with impaired growth hormone (GH) secretion, with possible negative repercussions on bone health. We aimed to investigate the relationships between GH secretory capacity, evaluated with GHRH + arginine stimulation test, and bone parameters, assessed with a dual-energy X-ray absorptiometer, in a population of adult female patients affected by overweight and obesity. **Methods**: We assessed 276 women affected by overweight or obesity referred to the High-Specialization Center for the Care of Obesity, Umberto I Polyclinic, between 2014 and 2019 with signs or symptoms of growth hormone deficiency (GHD). **Results**: A total of 97 patients were diagnosed with GHD, and 179 patients with normal GH secretion were considered our control group. GHD patients showed a significantly reduced trabecular bone score (TBS) (*p* = 0.01). Bone quality parameters corrected for body mass index (BMI) had a positive and significant linear correlation with stimulated GH secretory capacity. **Conclusions**: In conclusion, bone quality, evaluated by TBS and hip structural analysis, correlates with GH-stimulated secretory capacity. GHD may act as an additive factor in the alteration of bone microarchitecture in patients affected by obesity, who are already at a higher risk of fractures.

## 1. Introduction

Obesity is a multifactorial disease associated with a variety of metabolic and hormonal alterations [1,2], including reduced circulating growth hormone (GH) levels [3,4]. GH is produced by the adenohypophysis with a pulsatile rhythm, under the main stimulatory action of hypothalamic GHRH; it exerts its effects on adipose tissue, muscle, and bone both directly and through insulin-like growth factor-1 (IGF-1). In patients affected by obesity, GH secretory capacity may be reduced as a consequence of different pathophysiological mechanisms, such as the reduced amplitude of spontaneous secretory episodes in response to physiological stimuli, physical exercise, or sleep [5,6,7,8]. Adult patients with GHD show a complex clinical picture characterized by a reduction in the quality of life, alteration in body composition and metabolism, altered cardiac morphology and function, increased cardiovascular risk, and sleep disorders such as OSAS [9,10,11]. Many of these alterations are shared with people affected by obesity, and some of them may be at least partially reverted by weight loss or ameliorated with adequate dietary choices [12,13,14,15,16]. GHD is associated with a reduction in patients’ bone mineral density (BMD) [17,18,19,20,21,22]. Less is known about the effect of GHD on bone quality. Dual-energy X-ray absorptiometry (DXA) permits a good assessment of body composition, BMD, and bone microarchitecture through the trabecular bone score (TBS) and hip structural analysis (HSA) at the spine and hip levels, respectively. It is a reliable instrument, and it is more accessible and convenient and involves less ionizing radiation in comparison to the computed tomography scan (CT scan), which represents the gold standard [23,24].

This study aims to evaluate the relationships between GH secretory capacity, tested with the GHRH plus arginine test, and bone quantitative and qualitative parameters and body composition, assessed by DXA, in a population of women affected by overweight or obesity.

## 2. Materials and Methods

### 2.1. Patients

We evaluated 276 adult female outpatients affected by overweight or obesity (BMI ≥ 25 kg/m^2^) who were referred to our CASCO center (High-Specialization Center for the Care of Obesity), Umberto I Polyclinic, Sapienza University of Rome, between 2014 and 2019 and presented with at least one sign or symptom suggesting GH deficiency (reduced IGF-1 levels or at the lower limit of the normal range for age and sex, as well as fatigue, osteopenia, central adiposity, and dyslipidemia). Patients who were pregnant, breastfeeding, with active neoplastic diseases, or prescribed drugs known to affect pituitary function were excluded from this study.

All patients had their medical history collected and underwent a physical exam, laboratory exams, and a GHRH + arginine stimulation test as part of their diagnostic workup during hospitalization. All patients gave their written informed consent.

All procedures were performed in accordance with the ethical standards of the institutional and/or national research committee and with the 1964 Helsinki Declaration and its later amendments or comparable ethical standards. This study was reviewed and approved on 24 October 2019 by the Sapienza University of Rome Ethics Committee.

### 2.2. Anthropometric Measurements

Weight was measured to the nearest 0.1 kg and height to the nearest 0.1 cm. The patients wore light clothing and no shoes. Waist circumference (WC) was measured with non-stretchable tape over the unclothed abdomen just above the iliac crest at the end of normal expiration, and hip circumference (HC) was measured around the pelvis at the widest point. The tape was parallel to the floor and did not compress the skin. Waist-to-hip ratio (WHR) was calculated by dividing waist circumference by hip circumference and expressed as an absolute value. BMI was expressed as weight (kg)/height (m^2^). Overweight was defined by a BMI ≥ 25 kg/m^2^ and <30 kg/m^2^ and obesity by a BMI ≥ 30 kg/m^2^.

### 2.3. Laboratory Test

Blood samples were collected from fasting patients by venipuncture between 8 and 9 a.m. All patients underwent a stimulation test with GHRH + arginine, which was conducted by administering an intravenous bolus injection of 1 µg/kg of GHRH and 0.5 g/kg (until a maximum of 30 g) of arginine hydrochloride in a 30 min intravenous infusion. Blood samples were collected at 0 + 30, +45, and +60 min. Samples were then transferred to the local laboratory and handled according to the local standards of practice. GHD was diagnosed when the peak value was below 4.0 ng/mL in patients with obesity and 8 ng/mL in patients with overweight [25]. The area under the concentration–time curve (AUC) was calculated using the trapezoidal rule.

### 2.4. Dual-Energy X-ray Absorptiometry

All patients underwent dual-energy X-ray absorptiometry (DXA) (Hologic-Discovery A, software version 12.5.3:2, Marlborough, MA, USA) to study hip and lumbar spine parameters and total body composition. DXA was performed with subjects wearing light clothing and no shoes. The evaluated bone parameters included the lumbar spine from L2 to L4 and the hip T-score, total body (TB) bone mineral density (BMD), and lumbar spine and hip BMD, expressed in g/cm^2^. Osteoporosis was diagnosed when the T-score was equal to or below −2.5. TBS was calculated with the software TBS iNsight (Version 2.12, Med-Imaps, Pessac, France). Bone microarchitecture was considered normal when TBS ≥ 1.35, partially degraded when 1.2 < TBS < 1.35, and degraded when TBS ≤ 1.2 [26]. HSA was focused on the three sections of the narrow neck (NN), intertrochanter (IT), and femur shaft (FS), and for each of these, the analyzed parameters were Cross-Sectional Area (CSA) (expressed in cm^2^), Cross-Sectional Moment of Inertia (CSMI) (expressed in cm^4^), Section Modulus (Z) (expressed in cm^3^), Cortical thickness (Cort) (expressed in cm) and Buckling Ratio (BR), calculated as a ratio of the mean outer radius to the mean cortical thickness of the femoral neck (expressed as a ratio). Differently from the other HSA parameters, higher values of BR predict inferior strength.

For body composition, we considered the values of body fat or fat mass (FM) and lean mass or fat-free mass (FFM), as well as their respective values for the trunk, expressed both in absolute value (Kg) and percentage. Upper body Fat Deposition Index (UFDI) is the ratio between upper body fat (head, arm, and trunk fat expressed in kilograms) and lower body fat (legs fat in kilograms) and describes the central deposition of fat.

### 2.5. Statistical Analysis

Statistical analysis was performed using the software Statistica, version 12 StatSoft Inc. (Tulsa, OK, USA). In order to correct the parameters for BMI, we divided the parameter values of interest by BMI. All data are expressed as mean ± standard deviation (SD) unless otherwise specified. Normality was evaluated using the Shapiro–Wilk test. Comparison between the groups was made with Student’s *t*-test. Pearson correlations were made and considered statistically significant when *p* < 0.05.

## 3. Results

The general characteristics of our population are summarized in Table 1.

We diagnosed GH deficiency in 97 patients according to the peak values of the GHRH + arginine test. A total of 179 patients showed normal GH secretion and were considered our control group. Comparisons between the two groups for general characteristics, body composition, GH secretion, and bone parameters are shown in Table 2, Table 3 and Table 4. Basal IGF-1 did not significantly differ between GHD and control patients.

BMD values for the spine and hip overlap. Lumbar T-score does not significantly differ among groups, while hip T-score is significantly better in GHD patients. Regarding bone quality, TBS is significantly worse in GHD patients compared to the control group. On the other hand, confirming the T-score results, hip bone geometry evaluated with HSA is unexpectedly better in GHD patients for many femur shaft parameters.

Considering that mechanical load is a strong determinant of hip structural analysis parameters and that BMI, on the contrary, is a strong negative modulator of GH secretion, in order to highlight the true influence of GH secretory capacity on hip quality indices, we corrected DXA bone parameters for BMI, dividing bone parameters values by BMI. After correction for BMI, lumbar BMD, TBS, and HSA parameters were shown to be worse in GHD patients. See Table 5 for some bone parameters after correction for BMI.

Linear correlations between bone parameters and GH secretory capacity are shown in Table 6 and Table 7.

## 4. Discussion

Our cross-sectional study reveals a significant association between growth hormone secretory capacity and bone parameters in female individuals affected by overweight and obesity. Over 35% of our patients showed reduced GH secretory capacity, accompanied by a higher BMI and increased visceral fat. Despite comparable lumbar BMD, TBS indicated a worsened microarchitecture in the GH-deficient group and a partially degraded lumbar spine microarchitecture in the control group also. Unexpectedly, GH deficiency patients had higher hip BMD and preserved femur shaft architecture. However, correction for BMI reversed these associations, highlighting the influence of body mass on bone health.

Patients affected by obesity may have a reduced GH secretory capacity; in our cohort, more than 35% of patients had a condition of GHD. IGF-1 basal values did not statistically differ among GHD patients and the control group, and a trend for lower values in the GHD group was observed. This was expected, as IGF-1 synthesis depends on GH levels; it is the mediator of the action of GH and, in turn, regulates GH secretion [27]. Symptoms, even blurred ones, are extremely important in guiding the suspicion of GHD in adults. As expected, the patients in the GHD group had higher BMI, waist circumference, total fat mass, trunk fat and UFDI, suggesting a trend for more pronounced visceral fat mass accumulation in GHD patients.

The positive correlation between GH secretion and BMD is confirmed in studies in children; however, these patients often have multiple pituitary hormone deficiencies, and it is not completely clear whether the BMD reduction and the increased fracture risks are mainly due to GH deficiency or are the result of a more complex hormonal picture [28,29].

In our cohort, lumbar BMD did not significantly differ on the basis of GHD, while hip BMD was unexpectedly higher in GHD patients. Considering that GHD patients had a higher BMI, this finding may hypothetically be ascribed to the patients’ higher BMI. Other studies, in fact, found that women affected by overweight or obesity had higher hip BMD and superior hip geometry and strength as evaluated by HSA, and we observed a tendency in the same direction in our cohort, compared with controls [30]. In any case, the protective role against osteoporosis and bone fractures traditionally ascribed to obesity due to the positive correlation between BMI and BMD has been overcome thanks to recent evidence of increased hip fracture risks in patients affected by obesity, correlated with waist circumference and waist-to-hip ratio [31,32,33,34]. In fact, in our cohort, the above-mentioned positive association was reverted after correction for BMI. The negative effect of GHD on bone strength may therefore be an additive factor for fracture risk in patients affected by obesity. In previous studies about the correlation between bone health and GH secretory capacity, as expected, GHD patients had decreased BMD and an increased fracture risk [35,36,37,38,39].

Whether these alterations can be reverted by GH substitute therapy is still not clear, as not all studies invariably report beneficial effects of GH therapy on BMD and TBS [40,41,42,43,44,45]. A recent 10-year prospective follow-up study of 63 adult GHD patients who were administered GH replacement therapy reported an increase in lumbar and total hip BMD and, on the contrary, no significant changes in TBS [46].

GHD can negatively impact bone quality, considering the fundamental role of GH in bone growth and structure. The GH-IGF-1 axis acts on the remodeling of bone tissue through reducing the renal excretion of phosphates, stimulating vitamin D 1-α-hydroxylation and calcium intestinal absorption, and mediating parathyroid hormone action [47,48,49,50]. TBS was lower in the GHD group, indicating a degraded microarchitecture. In the control group, we observed a partially degraded architecture. The fact that, in both groups, lumbar BMD was nearly normal and TBS was at least partially degraded supports the theory that people affected by obesity have a degraded bone microarchitecture despite good BMD; therefore, only considering BMD as a fracture risk indicator in people with obesity is limiting and misleading.

Hip structural analysis showed that GHD patients had a more preserved femur shaft architecture, with better CSA, CSMI, and Z values. The microarchitecture of the femur is influenced by the mechanical load on the joint, which is strictly related to the weight of the patient.

GHD seemed to have a stronger impact on lumbar spine bone quality, expressed as TBS, than femur bone quality, expressed as HAS. Some interventional studies with GH replacement in GHD patients report that GH therapy has a greater effect in terms of increased bone density on the lumbar spine in comparison to the femur [51,52,53,54,55,56,57,58], and these results should be ascribed mainly to male patients [52,57,59]. The reason for this different impact on the lumbar spine and the femur is still unknown. We found only a few studies that investigated lumbar bone microarchitecture in GHD patients, and the effect of GHD substitutive therapy on TBS was not concordant among them [51,59,60]. An influence of 25(OH)D vitamin status on this is possible, because the amelioration induced by GH replacement therapy needs a concomitant vitamin D sufficiency [60].

The structural parameters that we used in our study are easily calculated using usual DXA images, without further ionizing radiation, and can better assess the patients’ fracture risk, which would be underestimated with the only evaluation of BMD. The use of DXA for HSA evaluation, although the gold standard is represented by the CT scan, is considered a valid compromise to obtain reliable data with lower exposition to ionizing radiation and lower cost using a relatively common software [23,24].

Among the limitations of our research is the cross-sectional nature of this study, which allows us to identify the associations between GH secretory capacity and bone parameters but does not allow us to define a causality relation. On the other hand, the single-center nature of our study minimizes inter-operator variability. Moreover, we opted for a gender-specific approach and focused on individuals with weight excess.

Our study emphasizes the importance of moving beyond traditional BMD measurements. TBS and hip structural analysis should be further evaluated in future research studies and considered for routine use in evaluating fracture risk. In fact, GHD may act as an additive factor in the alteration of bone microarchitecture in patients with weight excess, who are already at higher risk for fractures.

To the best of our knowledge, our research is the first to study bone quality parameters in female patients affected by weight excess and GHD.

## 5. Conclusions

Our findings reflect the intricate relationship between GH secretory capacity and bone quality. In fact, in our cohort, GHD patients had significantly reduced TBS. Lumbar BMD, TBS, and HSA parameters were worse in the GHD group after correction for BMI. Bone quality parameters corrected for BMI showed a positive linear correlation with stimulated GH secretory capacity. In conclusion, GH deficiency in female individuals with weight excess may influence bone quality, with potential implications for fracture risk.

## Figures and Tables

**Table 1:** General characteristics and body composition of our population, expressed as mean ± SD.

	Our Cohort (*n* = 276)
Age (years)	45.08 ± 12.56
Height (m)	1.61 ± 0.07
Weight (kg)	99.93 ± 21.60
BMI (kg/m^2^)	38.53 ± 7.81
WC (cm)	117.83 ± 16.59
HC (cm)	122.03 ± 13.66
WHR	0.95 ± 0.09

BMI: body mass index; WC: waist circumference; HC: hip circumference; WHR: waist-to-hip ratio.

**Table 2:** General characteristics and body composition parameters according to GH secretory capacity expressed as mean ± SD.

Demographic, Anthropometric, and Body Composition Parameters	Controls (*n* = 179)	GHD (*n* = 97)	*p* Value
Age (years)	45.25 ± 13.69	46.81 ± 10.13	0.324
Height (m)	1.61 ± 0.07	1.61 ± 0.07	0.662
Weight (kg)	95.69 ± 18.12	107.74 ± 25.15	0.000
BMI (kg/m^2^)	36.81 ± 6.25	41.71 ± 9.30	0.000
WC (cm)	113.87 ± 14.88	125.07 ± 17.19	0.000
HC (cm)	120.92 ± 13.32	125.17 ± 14.28	0.014
WHR	0.94 ± 0.09	0.97 ± 0.10	0.011
FM %	42.06 ± 5.04	42.70 ± 5.71	0.362
FFM %	57.94 ± 5.04	57.29 ± 5.71	0.362
FM (kg)	39.81 ± 10.66	42.98 ± 10.91	0.029
FFM (kg)	53.77 ± 8.85	56.72 ± 9.49	0.016
Trunk Fat (kg)	18.70 ± 5.89	20.90 ± 5.78	0.005
Trunk Fat (%)	39.74 ± 5.85	41.22 ± 6.63	0.074
Trunk Lean (kg)	27.19 ± 4.77	28.70 ± 4.98	0.020
UFDI	1.75± 0.52	1.93 ± 0.60	0.010

BMI: body mass index; WC: waist circumference; WHR: waist-to-hip ratio; FM: fat mass; FFM: fat-free mass; UFDI: Upper body Fat Deposition Index.

**Table 3:** GH secretory capacity in controls and GHD patients, expressed as mean ± SD.

Parameter	Controls (*n* = 179)	GHD (*n* = 97)	*p* Value
AUC GH after GHRH + Arg (ng/mL/h)	524.39 ± 389.13	119.69 ± 69.72	0.000
Peak (ng/mL)	17.07 ± 12.47	3.48 ± 1.85	0.000
IGF-1 (ng/mL)	145.95 ± 68.15	133.89 ± 55.31	0.136

AUC: area under the concentration–time curve.

**Table 4:** Bone parameters obtained by DXA in controls and GHD patients, expressed as mean ± SD.

Bone Parameters	Controls (*n* = 179)	GHD (*n* = 97)	*p* Value
Lumbar T-score	−0.07 ± 1.43	−0.07 ± 1.51	0.994
Lumbar BMD (g/cm^2^)	1.04 ± 0.153	1.05 ± 0.16	0.772
Hip T-score	0.42 ± 1.19	0.77 ± 1.21	0.037
Hip BMD (g/cm^2^)	0.99 ± 0.15	1.02 ± 0.16	0.103
TBS	1.27 ± 0.14	1.21 ± 0.15	0.011
TB BMD (g/cm^2^)	1.09 ± 0.12	1.13 ± 0.12	0.022
NN CSA (cm^2^)	3.20 ± 0.55	3.19 ± 0.58	0.901
NN CSMI (cm^4^)	2.87 ± 0.74	2.81 ± 0.96	0.630
NN Z (cm^3^)	1.53 ± 0.33	1.49 ± 0.39	0.552
NN Cort (cm)	0.19 ± 0.04	0.19 ± 0.04	0.872
NN BR	10.36 ± 2.83	10.28 ± 2.94	0.885
IT CSA (cm^2^)	5.51 ± 1.05	5.83 ± 1.25	0.073
IT CSMI (cm^4^)	14.82 ± 4.19	15.99 ± 5.89	0.129
IT Z (cm^3^)	4.72 ± 1.15	5.07 ± 1.47	0.087
IT Cort (cm)	0.44 ± 0.09	0.45 ± 0.10	0.439
IT BR	7.38 ± 1.74	7.16 ± 1.63	0.433
FS CSA (cm^2^)	4.62 ± 0.79	4.89 ± 0.89	0.043
FS CSMI (cm^4^)	4.04 ± 1.11	4.48 ± 1.46	0.029
FS Z (cm^3^)	2.55 ± 0.53	2.74 ± 0.66	0.038
FS Cort (cm)	0.61 ± 0.12	0.62 ± 0.12	0.463
FS BR	2.66 ± 0.63	2.68 ± 0.62	0.868

BMD = bone mineral density; TBS = trabecular bone score; TB BMD = total body BMD; NN = narrow neck; IT = intertrochanter; FS = femur shaft; CSA = Cross-Sectional Area; CSMI = Cross Sectional Moment of Inertia; Z = Section Modulus; Cort = Cortical thickness; BR = Buckling Ratio.

**Table 5:** Ratio between bone parameters and BMI (gr/cm^2^) obtained with DXA in controls and GHD patients, expressed as mean ± SD.

Bone Parameters/BMI	Controls (*n* = 179)	GHD (*n* = 97)	*p* Value
Lumbar BMD (g/cm^2^)/BMI(g/cm^2^)	0.29 ± 0.06	0.27 ± 0.05	0.004
TBS/BMI (g/cm^2^)	0.36 ± 0.08	0.31 ± 0.08	0.001
Hip BMD (g/cm^2^)/BMI (g/cm^2^)	0.27 ± 0.05	0.26 ± 0.04	0.030
**NN CSA (cm^2^)/BMI (g/cm^2^)**	0.034 ± 0.01	0.031 ± 0.01	0.012
NN Cort/BMI (cm)/BMI (g/cm^2^)	0.05 ± 0.01	0.049 ± 0.01	0.011
IT CSA (cm^2^)/BMI (g/cm^2^)	1.52 ± 0.27	1.50 ± 0.34	0.569
IT Cort (cm)/BMI (g/cm^2^)	0.12 ± 0.03	0.11 ± 0.02	0.072
FS CSA (cm^2^)/BMI (g/cm^2^)	1.27 ± 0.19	1.25 ± 0.24	0.439
FS Cort (cm)/BMI (g/cm^2^)	0.17 ± 0.03	0.16 ± 0.03	0.026

NN = narrow neck; IT = intertrochanter; FS = femur shaft; CSA = Cross-Sectional Area; Cort = Cortical thickness.

**Table 6:** Pearson correlation between bone parameters and GH secretion, measured as AUC GH after GHRH + arginine test (ng/mL/h) before and after correction for BMI (g/cm^2^) (*n* = 190). Correlations are expressed as Pearson correlation coefficients.

Bone Quality Parameters	Before Correction for BMI		After Correction for BMI	
	r	*p* Value	r	*p* Value
TBS	0.253	0.000	0.414	0.000
NN CSA (cm^2^)	−0.178	0.006	0.233	0.000

TBS: Trabecular bone score; NN = narrow neck; CSA = Cross-Sectional Area.

**Table 7:** Pearson correlation among the ratio between hip parameters and BMI, corrected for age, and GH secretion, measured as AUC GH after GHRH +arginine test (ng/mL/h) (*n* = 190). Correlations are expressed as Pearson correlation coefficients.

DXA Parameter/BMI	r	*p* Value
Lumbar BMD (g/cm^2^)/BMI (gr/cm^2^)	0.262	0.000
Hip BMD(g/cm^2^)/BMI (gr/cm^2^)	0.205	0.000
TBS (g/cm^2^)/BMI (gr/cm^2^)	0.414	0.000
**NN CSA (cm^2^) (g/cm^2^)/BMI (gr/cm^2^)**	0.233	0.000
NN Cort (cm) (g/cm^2^)/BMI (gr/cm^2^)	0.199	0.005
IT CSA (cm^2^) (g/cm^2^)/BMI (gr/cm^2^)	0.089	0.222
IT Cort (cm) (g/cm^2^)/BMI (gr/cm^2^)	0.183	0.010
FS CSA (cm^2^) (g/cm^2^)/BMI (gr/cm^2^)	0.053	0.467
FS Cort (cm) (g/cm^2^)/BMI (gr/cm^2^)	0.140	0.050

NN = narrow neck; IT = intertrochanter; FS = femur shaft; CSA = Cross-Sectional Area; Cort = Cortical thickness.

## Data Availability

Data are available from the corresponding author upon reasonable request.
